# The Pain Frequency-Severity-Duration Scale as a Measure of Pain: Preliminary Validation in a Pediatric Chronic Pain Sample

**DOI:** 10.1155/2014/653592

**Published:** 2014-01-20

**Authors:** Katherine S. Salamon, W. Hobart Davies, Melissa R. Fuentes, Steven J. Weisman, Keri R. Hainsworth

**Affiliations:** ^1^Division of Pain Medicine, Children's National Medical Center, 111 Michigan Ave, NW, Washington, DC 20010, USA; ^2^University of Wisconsin-Milwaukee, 224 Garland Hall, 2441 Hartford Ave, Milwaukee, WI 53211, USA; ^3^Rogers Memorial Hospital, 11101 Lincoln Ave, Milwaukee, WI 53227, USA; ^4^Children's Hospital of Wisconsin and Medical College of Wisconsin, 9000 W. Wisconsin Ave, Milwaukee, WI 53226, USA

## Abstract

Typically, pain is measured by intensity and sensory characteristics. Although intensity is one of the most common dimensions of pain assessment, it has been suggested that measuring pain intensity in isolation is only capturing part of the pain experience and may not lead to an accurate measurement of how pain impacts a child's daily functioning. The current study aimed to develop a measure that would capture pain intensity along with frequency and duration in a clinical sample of youth diagnosed with chronic pain. The pain-frequency-severity-duration (PFSD) scale was developed and data were collected from a multidisciplinary pain clinic at a large, midwestern children's hospital. Validated measures of functional limitations and health related quality of life were also collected. Significant correlations were found between the PFSD composite score, functional limitations, and health related quality of life. Future research should continue to evaluate this questionnaire utilizing other validated pain measures and other areas potentially impacted by chronic pain and with more diverse samples. This initial finding suggests that the PFSD is a convenient self-reported measure and is strongly related to health related quality of life and functional disability.

## 1. Introduction

Idiopathic chronic pain is persistent pain, lasting longer than three months, that does not serve as a somatic warning sign of tissue damage or injury [1]. It is estimated that one in five children in the United States is affected by chronic pain [1]. Typically, pain is measured by its intensity and sensory characteristics (e.g., location and pattern, including frequency and duration) [2]. Specifically, pain frequency, severity/intensity, and duration are often assessed simultaneously at medical appointments and during hospital visits. Self-reported pain intensity continues to be the most widely used measurement of a child's pain [3]. Although intensity is the most common dimension of pain assessment, von Baeyer has suggested that measuring pain intensity alone is only capturing part of the clinical picture [4, 5]. Given that pain is typically thought of as a subjective experience, having an accurate measurement of pain intensity does not guarantee an accurate measurement of how that pain impacts a child's daily functioning. For example, two youths may report similar intensity of pain on a 10 point scale; however, one youth may limit social and physical involvement while the other continues to engage in daily activities with minimal impairment. Therefore, it is important to have a pain measure that better reflects the whole pain experience.

Youth with chronic pain can have decreased social functioning, increased school absenteeism, and decreased quality of life [6, 7]. Standard assessment of pain intensity and duration has notoriously been unhelpful in the past as it is not predictive of functioning. Research in pediatric chronic pain has found inconsistencies within the relationship between pain intensity/duration and functional outcomes, such as disability and quality of life [8], indicating that assessing for intensity and duration of pain may not provide an accurate representation of the pain experience for a particular youth. Wendland and colleagues [9] noted that research on pain intensity and duration has only predicted a moderate amount of variance in functional outcomes. Therefore, assessing pain intensity and duration only captures one part of the whole pain experience and may not be useful in understanding quality of life or functional disability. Research has also noted that the relationship between pain, pain symptoms, and functional disability is not linear [9]. For example, pain symptoms, child's anxiety, and child's depression were all implicated in the presentation of functional disability and the authors suggested that the degree of disability is likely influenced by numerous factors. Given the complicated picture of chronic pain and the impact of functional disability, it is important to develop valid measures of pain frequency, severity, and duration that focus on daily functioning, as well as pain intensity to accurately treat these populations.

The purpose of the current study was to develop and validate a pain assessment tool that incorporated multiple aspects of the pain experience, in order to better assess the impact on daily functioning. Specifically, this includes elaborated measures of pain that are informative about the intrusiveness, or impact on daily functioning, of pain. As the experience of chronic pain can lead to numerous social, academic, emotional, and physical limitations, it is important to have a pain scale that can reliably assess pain and provide more predictive information on the impact of the pain in the youth's daily activities and health related quality of life. The pain-frequency-severity-duration (PFSD) scale was designed to assess not only the intensity of pain, as has been done in the past, but also to assess the intrusiveness that chronic pain has on the youth's life. It was hypothesized that the PFSD composite score would be related to worst and usual pain level reported during the clinic appointment. Secondly, it was hypothesized that the PFSD composite score would be more significantly related to activity limitations and health related quality of life, as compared to pain intensity alone.

## 2. Materials and Methods

### 2.1. Participants

Three hundred and fifty three youth seen in a multidisciplinary pain clinic from December 2009 through February 2011 completed the questionnaires. All families referred to the pain clinic received questionnaires prior to the initial appointment. Information about participants who did not complete the questionnaires is not available as completion of the questionnaires is voluntary. Of those who did complete the questionnaires related to this study (*n* = 278), about 69% were female, 77% were Caucasian, and the average age was 14.07 years (SD = 2.64). These characteristics reflect the typical patient treated in the clinic. The top three reported pain locations were head (37%), abdomen (16.0%), and back (16.0%). The majority (96%) received medication as part of their treatment. About 80% (*n* = 264) received a mental health diagnosis (e.g., adjustment disorder, anxiety disorder, and depressive disorder) and therapy was recommended for 78%. Most (59.4%) reported pain lasting longer than one year. See Table 1 for more information about the demographic characteristics of the sample.

### 2.2. Measures

The *pain-frequency-severity-duration scale* (PFSD, see the Appendix) was developed in an effort to assess multiple aspects of pain and to broaden the focus to capture more than pain intensity. The PFSD was designed for youth ranging from 8–18 years of age and consists of five questions. The first question asked that participants circle the number of days in the past two weeks that they have experienced pain (0–14). The second and fourth questions asked that youth rate usual and worst pain intensity over the last two weeks using a Likert scale ranging from 0 (no pain) to 10 (worst pain). Questions three and five assessed the average duration of usual and worst pain by asking youth to indicate the length of pain in hours: 1-2, 3–5, 6–8, 9–12, 12–18, and 18–24. A composite score was derived by multiplying the number of days of pain, the level of usual pain, and the level of worst pain and then dividing the product by 10. For example, if a youth reported 10 days of pain over the 14 day period, a level 6 out of 10 usual pain level, and 9 out of 10 worst pain level, that participant's PFSD score would be 54.

The *child activity limitations questionnaire* (CALQ) is a validated [10] self-report measure which assesses functional disability over the last four weeks. Respondents rated the difficulty of performing 21 activities (e.g., going to school, playing with friends, etc.) on a six-point Likert scale from 0 (not at all difficult) to 5 (extremely difficult). A total score was utilized for the current study which can range from 0 to 105, where higher scores represent greater activity limitations (*α*_current sample_ = 0.95).

The *pediatric quality of life inventory, v.4.0 *(PedsQL 4.0) [11] is a well-validated 23-item self-report measure which assesses health related quality of life. Respondents rated perceived impact of the pain and the treatment on a variety of functional domains (physical, emotional, social, and school functioning) on a five-point Likert scale ranging from 0 (never) to 4 (almost always). Items were scored according to the developer's instructions. A total score was calculated, which can range from 0 to 100, with higher scores indicating better health related quality of life (*α*_current sample_ = 0.87). Subscale scores were also utilized, including the physical composite (*α*_current sample_ = 0.70), the psychosocial composite (*α*_current sample_ = 0.85), emotional summary (*α*_current sample_ = 0.77), social summary (*α*_current sample_ = 0.19), and school summary (*α*_current sample_ = 0.75).

*Demographic information *from the deidentified database included information on age, gender, ethnicity, and the pain clinic's diagnosis and whether mental health services were recommended. Worst and usual pain were assessed by the physician during the initial appointment by asking youth to indicate pain over the last one to two weeks on a 0 (no pain)–10 (worst pain imaginable) point-Likert scale.

### 2.3. Procedure and Data Analysis

Youth who presented to a multidisciplinary pain clinic at a large, midwestern children's hospital completed several questionnaires as part of the initial intake appointment. Clinicians utilized these measures for assessment and treatment planning. Families were aware that the questionnaires would be de-identified and used for research purposes. The study received hospital IRB approval for the retrospective chart review.

Demographic characteristics of the sample and descriptive information of the variables under study were provided (see Table 1). Cronbach's alphas were calculated for each of the measures. Pearson correlations and *t*-tests were utilized to determine the need to control for covariates. The relationship between the PFSD composite score and activity limitations and health related quality of life was explored utilizing partial correlations. All analyses were conducted using SPSS 17.

## 3. Results

The mean for the total self-reported CALQ and the total self-reported PedsQL indicated that the participants were experiencing at risk levels of pain related impact on their daily lives [10, 12].

### 3.1. PFSD Individual Items

Reported pain frequency over 2 weeks ranged from 0 to 14 days with a mean of 10.99 days. Usual pain intensity averaged 6.46 of 10 (*n* = 282, SD = 2.04) and worst pain intensity averaged 8.29 of 10 (*n* = 284, SD = 1.92). About one third (33.6%) reported usual pain duration of 18*‒*24 hours, with 3–5 hours per day as the next most endorsed duration category. About one third (29.1%) reported worst pain duration as 3–5 hours, with 1-2 hours as the next most endorsed category.

### 3.2. PFSD Composite Score

**Figure 1:**
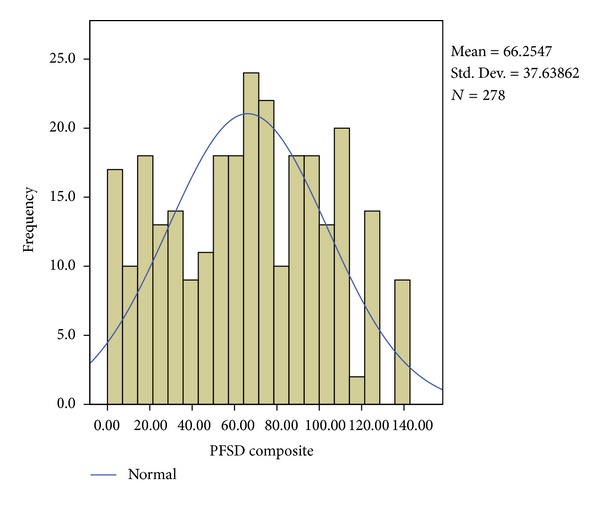
Histogram of PFSD composite scores with normal curve.

A subset of patients (*n* = 278) from the larger sample completed the PFSD. PFSD composite scores, which can range from 0–140, were normally distributed (Kolmogorov-Smirnov *Z* = 1.22, *P* = 0.101; see Figure 1) with an average score of 66.25 (SD = 37.64). The PFSD composite score was not significantly associated with age or ethnicity. However, it did differ significantly across gender (*t*(276) = −3.72, *P* = 0.000), with females reporting higher PFSD composite scores (M = 71.80, SD = 37.25) as compared to males (M = 54.08, SD = 35.76). Therefore, gender was used as a covariate in the following analyses.

When controlling for gender, the PFSD individual items were significantly associated with worst pain and usual pain intensity reported during the clinic appointment. Correlations among the PFSD, worst and usual pain assessed at the appointment, CALQ total, and PedsQL total and subscale scores were all significant (Table 2). However, the correlations between worst and usual pain and the measures of pain related impairment (CALQ and PedsQL) were lower than those between the PFSD individual items and composite scores. Pearson correlations were utilized to determine if the PFSD composite score was a better predictor of activity limitations and health related quality of life as compared to the three individual components of the PFSD composite score. As seen in Table 2, the PFSD composite was observed to be more significantly related to all domains of functioning as compared to the individual components of the score. The PFSD Composite score offered only marginal improvement in the prediction of functional disability and health-related quality of life, especially compared to the PFSD worst pain rating.

## 4. Discussion

The current study was designed to develop a self-reported pain questionnaire that could capture the multidimensionality of the pain experience, as well as describe the intrusiveness of pain on daily functioning. Unlike what has been found using pain intensity alone, significant correlations were found between the PFSD composite score and pain intensity assessed during the clinic appointment, with both activity limitations and health related quality of life. The initial results suggest that the PFSD is a potentially promising self-report measurement and may be an improved assessment of the impact of pain on functioning than isolated pain dimensions.

The PFSD incorporates multiple aspects of pain, in order to better capture the impact of the pain experience and the PFSD composite score is thought to be more reflective of the overarching experience on the youth's life than assessing pain intensity in isolation. It is important to note that the standard 10-point pain scale assessing usual and worst pain is imbedded within the measure and could be used separately. The PFSD includes other questions to compute a composite score in order to assess the impact of pain beyond intensity.

Huguet et al. [13] have recently provided several recommendations for specific pain intensity scales that have been validated for different age ranges, but there remains a paucity of research on measures that links both pain intensity and functional disability. Clinically, utilizing one measure to accurately predict the impact of pain could provide important treatment information. Rather than having a patient complete several measures, the current results indicate a single measure may provide a quick assessment to help inform treatment recommendations and delineate the global significance of pain for the patient. The PFSD may also decrease participant burden while still providing key information regarding the pain experience.

It is important to note that all of the individual components of the PFSD are better predictors of functional disability and health-related quality of life than pain ratings made verbally in the clinical setting. The PFSD composite appears to offer only marginal predictive improvement over the PFSD worst pain rating, at least in this sample of youth with complex chronic pain. The information from the other components of the PFSD may be relevant to research or practice and may contribute to more unique variance in a more heterogeneous population. Further research should address this and explore potential implications. As all of the youth in this sample presented with complex chronic pain, research with the PFSD in more acute pain settings as well as youth presenting with less complex pain presentations is necessary.

One limitation of the current study was that the sample was relatively homogeneous. Although this sample closely represented those who present to multidisciplinary pain clinics, more research is needed to determine whether the PFSD is valid for ethnically and culturally diverse groups. Another limitation was the reliance on self-report. It may be beneficial to collect data from multiple reporters, such as caregivers and siblings. Future research is needed for the development of a parent proxy measure as well as determination of the predictive value of the PFSD for both clinical practice and research. First, a parent proxy version could be used in situations when the child is too young or cognitively impairment and unable to provide information about pain intensity and functional disability. Currently, the literature is lacking in assessment for younger and cognitively impaired youth [13, 14], and utilizing one measure for multiple purposes would decrease the burden on the parents and simplify clinical reporting. Secondly, a measure of pain intensity that also reflects pain's impact may serve as a useful clinical tool when treating these populations to aid in bringing both the parent and youth together for treatment planning. A final limitation is that the timeframe for the measures, specifically the pain report and self-reported QOL, was not consistent. It may be beneficial for further studies to utilize reports that make use of the same timeframe in order to determine if this is a confounding factor in the current study.

Although it was not possible to compare other validated pain measures with the PFSD in the current study, future research is required to determine whether the PFSD composite score is significantly associated with data collected from validated pain measures, as well as objective measures of functioning. Given the novel approach to studying pain intensity and functional impact, it is expected that the PFSD will be associated with pain on validated measures. Future studies that include validated measures of pain and functional limitations, health related quality of life, and other aspects of the pain experience will be essential to compare the relationships between these variables and the PFSD. Future studies are also needed to validate the PFSD for varying pain diagnoses.

## 5. Conclusion

While research supports that self-reported pain intensity is a valid way of assessing presence and intensity of pain [13], a scale that includes frequency, severity, and duration together may provide a convenient way to assess the multidimensionality of pain and be able to capture the full pain experience through increased prediction of functional impact. Pain is often assessed in a variety of settings and under different circumstances [15]. Therefore, simple, convenient, and valid measures of pain are required to best assess and treat pain in acute and chronic care settings. The results of the current study suggest that the PFSD may be such a measure.

## Figures and Tables

**Table 1:** Demographic characteristics and descriptive information (*n* = 278).

	*N*	%	M	SD	Range
Age (years)			14.07	2.64	8–18
Gender					
Female	191	69.0%			
Male	87	31.0%			
Ethnicity					
Caucasian	215	77.0%			
African-American	26	9.0%			
Multiracial	11	4.0%			
Hispanic	14	5.0%			
American Indian	1	<1%			
Missing/not reported	11	4.0%			
Pain location					
Head	99	37.0%			
Abdomen	44	16.0%			
Back	44	16.0%			
Lower extremity	33	12.0%			
Upper extremity	17	6.0%			
Generalized	15	6.0%			
Other	17	6.0%			
Usual pain (clinic visit)			5.86	2.36	0–10
Worst pain (clinic visit)			8.55	1.46	0–10
PFSD composite score			66.26	37.64	0–140
CALQ total score			41.06	22.63	0–101
PedsQL total score			57.56	17.28	4.35–100
Physical composite			48.86	23.96	0–100
Psychosocial composite			61.64	17.27	6.67–100
Emotional summary			58.40	22.03	0–100
Social summary			76.06	20.29	5–100
School summary			50.36	23.40	0–100

CALQ: child activity limitations questionnaire. PFSD: pain-frequency-severity-duration. PedsQL: pediatric quality of life.

**Table 2:** Bivariate and partial correlations among variables under study.

	Usual pain (clinic visit)		Worst pain (clinic visit)		PFSD composite^a^		PFSD days of pain		Usual pain (PFSD)		Worst pain (PFSD)	
	*r*	*P*	*r*	*P*	*r*	*P*	*r*	*P*	*r*	*P*	*r*	*P*
CALQ total	0.23	0.000***	0.24	0.000***	0.49	0.000***	0.40	0.000***	0.43	0.000***	0.49	0.000***
PedsQL total	−0.23	0.000***	−0.25	0.000***	−0.38	0.000***	−0.29	0.000***	−0.32	0.001***	−0.36	0.000***
PedsQL physical	−0.21	0.001***	−0.19	0.004***	−0.40	0.000***	−0.37	0.000***	−0.30	0.000***	−0.37	0.000***
PedsQL psychosocial	−0.21	0.001***	−0.24	0.000***	−0.30	0.000***	−0.18	0.003***	−0.27	0.000***	−0.28	0.000***
PedsQL emotional	−0.21	0.002***	−0.24	0.000***	−0.28	0.000***	−0.19	0.001***	−0.26	0.000***	−0.24	0.000***
PedsQL social	−0.09	NS	−0.13	0.043**	−0.21	0.001***	−0.13	0.028**	−0.20	0.001***	−0.19	0.001***
PedsQL school	−0.20	0.002***	−0.21	0.001***	−0.22	0.001***	−0.09	NS	−0.17	0.005***	−0.23	0.000***

^a^Partial correlations were used to control for gender. **P < 0.05. ***P < 0.01. *n* = 235. NS: not significant. CALQ: child activity limitations questionnaire. PFSD: pain-frequency-severity-duration. PedsQL: pediatric quality of life.

## Appendix

### Pain Frequency-Severity-Duration Scale

Please think about the pain symptoms you have experienced over the last two weeks. Please answer the following questions based in this experience.

(1) About how many days in the past 2 weeks have you been in pain?
**Table:**
  1	2	3	4	5	6	7
  8	9	10	11	12	13	14
If you answered 0 to question 1, please stop here.
(2) About how long have you had a pain problem? …
(3) Would you describe your pain as recurrent or continuous (circle one)?

Please think about your *usual* or typical level of pain on the days that you have had pain in the last 2 weeks.

(4) On the days that you have been in pain, what has been your *usual* level of pain (0 = No pain; 10 = Worst pain you can imagine)
**Table:**
  0	1	2	3	4	5	6	7	8	9	10
  No Pain										Worst Pain
(5) On average, how many *hours* has this *usual* pain lasted?
**Table:**
  1-2	3–5	6–8	9–12	12–18	18–24
Now think about the days that you had your *worst* level of pain in the last 2 weeks.
(6) On the days that you have been in pain, what has been your *worst* level of pain (0 = No pain; 10 = Worst pain you can imagine)
**Table:**
  0	1	2	3	4	5	6	7	8	9	10
  No Pain										Worst Pain
(7) On average, how many *hours* has this *worst* pain lasted?
**Table:**
  1-2	3–5	6–8	9–12	12–18	18–24
